# Atypical cutaneous leishmaniasis: a new challenge to VL elimination in South-East Asia

**DOI:** 10.3389/fcimb.2024.1454002

**Published:** 2024-11-04

**Authors:** Manju Jain, Diya A’gitok Sangma, Lipsalely Parida, Rohit Negi, Ajeet Negi, Greg Matlashewski, Patrick Lypaczewski

**Affiliations:** ^1^ Department of Biochemistry, Central University of Punjab, Bathinda, Punjab, India; ^2^ Department of Dermatology, Mahatma Gandhi Medical Services Complex, Shimla, Himachal Pradesh, India; ^3^ Department of Dermatology, Indira Gandhi Medical College and Hospital, Shimla, Himachal Pradesh, India; ^4^ Department of Microbiology and Immunology, McGill University, Montreal, QC, Canada

**Keywords:** Atypical, cutaneous leishmaniasis, *Leishmania donovani*, VL-elimination, South East Asia

## Abstract

Visceral leishmaniasis (VL) caused by *L. donovani* in South-East Asian endemic countries including India, Nepal and Bangladesh has been the primary focus of the ongoing VL elimination program. With a major reduction in VL cases resulting from the elimination program during the last two decades, the efforts are now focused on the challenges posed by potential reservoirs within the asymptomatic cases, HIV-co-infection VL cases and Post Kala-azar Dermal Leishmaniasis (PKDL) cases that continue to sustain the parasite transmission cycle in known and newer endemic zones. This article brings attention to a new potential parasite reservoir in the form of atypical cutaneous leishmaniasis (ACL) cases caused by novel *L. donovani* genetic variants. *L. donovani* mediated ACL is an emerging phenomenon in recent endemic sites that now justify a need for implementing molecular surveillance tools to identify region-specific *L. donovani* variants with dermotropic capabilities and potential to revert to visceral disease. A timely detection of novel ACL causing *L. donovani* genetic lineages in South-East Asian endemic regions is necessary to halt the spread of ACL and is potentially crucial for the sustainability of the advances made by the VL elimination.

## Introduction

Leishmaniasis is among the most neglected infectious tropical diseases and is caused by an intracellular protozoan parasite belonging to the genus *Leishmania*. More than 20 *Leishmania* species are prevalent in different parts of Africa, Central-South America, Mediterranean regions, Southern Europe and South-East Asia. *Leishmania* species-specific disease manifestation range from fatal systemic visceral leishmaniasis (VL) also known as Kala-Azar caused by the *L. donovani/L. infantum* complex, cutaneous leishmaniasis (CL) caused by *L. tropica/L. major* species in the Old World and mucocutaneous leishmaniasis (MCL) and CL caused by the *L. Viannia* subgenus in the New World (Alvar et al., 2012; World Health Organization, 2023). Due to the fatal nature of VL caused by *L. donovani* with a significant global burden, operational efforts are ongoing toward VL disease control and elimination. In 2005, with a share of ~70% in the global VL burden in South-East Asia, a disease elimination initiative was formulated by the WHO for the elimination of VL by 2015 in India, Bangladesh and Nepal with the objective to reduce the number of cases to less than one per 10,000 population in all endemic districts (World Health Organization, 2015). Elimination efforts included improved case detection, case management including rapid diagnosis and treatment, vector control and community education that have resulted in a dramatic reduction in VL cases in South-East Asia to less than 20% of the global burden (World Health Organization, 2022). Notwithstanding this progress, challenges remain including the emergence of novel *L. donovani* lineages in new areas acting as potential reservoirs for future outbreaks that could threaten the sustainability of the VL elimination program as discussed within.

## VL elimination targets and the challenges ahead

The VL elimination target in South-East Asia countries is defined as an annual incidence of less than one case per 10,000 population in endemic regions. On this scale, 99% of implementation units are reported to achieve the elimination threshold (Yajima et al., 2023). Among the VL endemic countries in the region, India reports a dramatic decrease in VL cases from 9241 in 2014 to 26 cases in 2024 (National Center for Vector Borne Diseases Control, 2024). Nepal reached the elimination target in 2013 although the emergence of several new VL cases in previously non-endemic low population districts breached the one case per 10,000 threshold (Epidemiology and Disease Control Division, 2019; Shrestha et al., 2019; Banjara and Joshi, 2020; Pandey et al., 2023). Bangladesh reached and sustained the elimination target for 3 years in all endemic districts in 2023 and has now entered the maintenance phase (World Health Organization, 2024). India is close to reaching the threshold. Bhutan and Thailand document sporadic cases in different districts and continue to accelerate case detection surveillance programs for disease elimination (Yangzom et al., 2012; Leelayoova et al., 2017; World Health Organization, 2021a; Dorji et al., 2024). Reaching the last remaining elimination target in South-East Asia by 2026 is supported by a regional strategic framework in alignment with the new neglected diseases elimination roadmap, 2021-2030 by the WHO (World Health Organization, 2021a, 2022).

**Figure 1:**
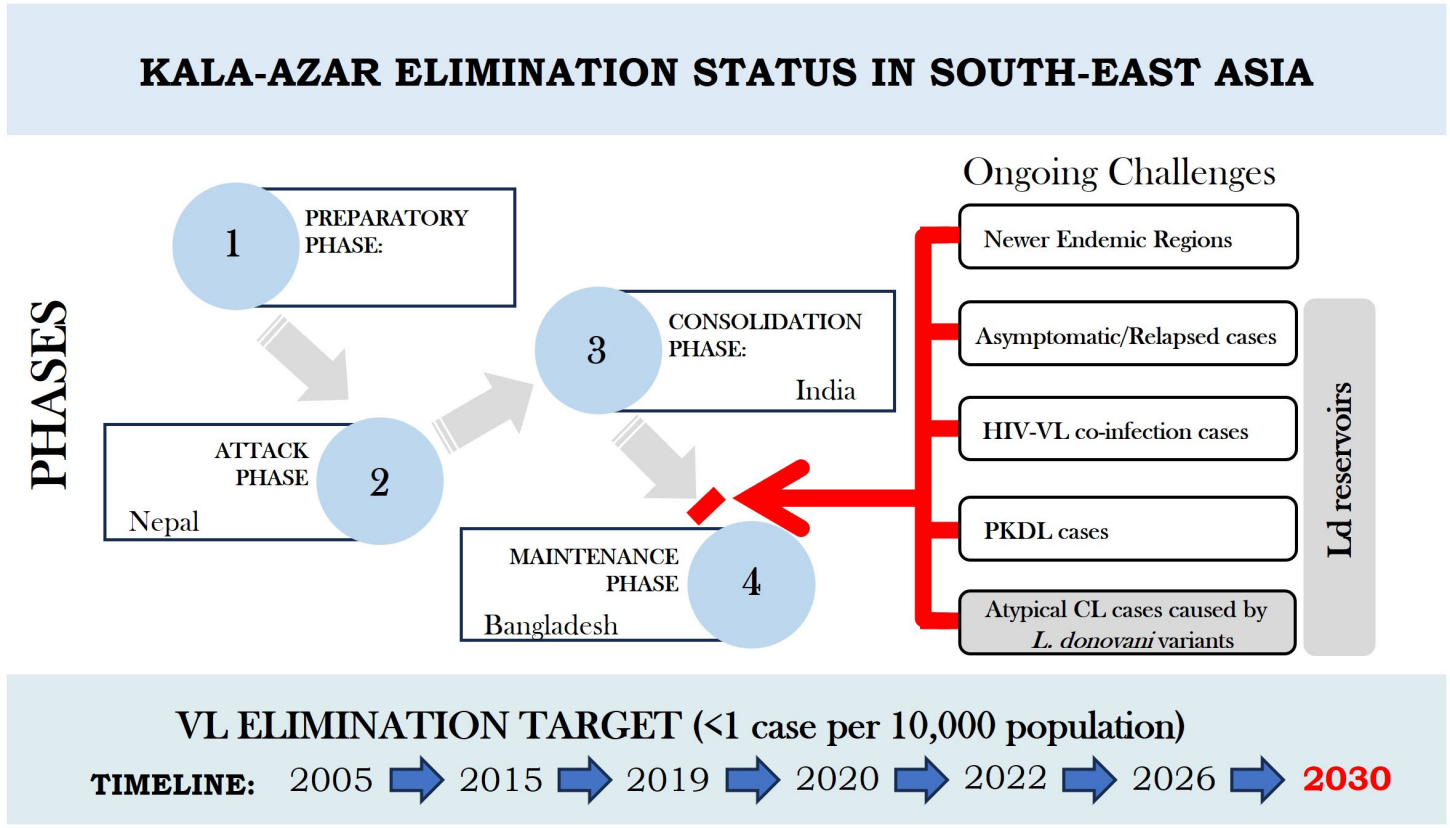
Current Scenario of Kala-azar Elimination programme in the Indian Subcontinent with extended timelines. Country specific phases achieved as per elimination targets are shown in terms of different phases viz preparatory phase, attack phase, consolidation phase and maintenance phase. Ongoing challenges in the way of India to move from consolidation phase to maintenance phase are indicated by red arrow and bar. The challenges also imply to Nepal to move further to achieve elimination target and to Bangladesh to maintain status of VL elimination through an integrated operational approach.

Challenges to the VL elimination program remain and include the emergence of new endemic sites, the continued presence of Post Kala-azar Dermal Leishmaniasis (PKDL) and asymptomatic cases as potential reservoirs, the emergence of HIV-co-infection cases and more recently atypical cutaneous leishmaniasis (ACL) cases as depicted in **Figure 1** (Manomat et al., 2017; World Health Organization, 2021a, 2022; Singh and Sundar, 2022; Ruang-Areerate et al., 2023; National Center for Vector Borne Diseases Control, 2024). Consequently, VL elimination programs have recognized the need for mandatory reporting of HIV-VL and PKDL cases and a need to develop strategies to identify asymptomatic cases for parasite detection in low disease burden settings (World Health Organization, 2021b, 2022; Singh and Sundar, 2022; Kumar et al., 2023; Ruang-Areerate et al., 2023). The availability of a *L. donovani* antigen leishmanin skin test (LST) to identify people previously infected will be important to support surveillance of transmission caused by asymptomatic and PKDL cases (Dey et al., 2023).

Despite the reduction in VL cases approaching the elimination target, VL cases from newer pockets in previously non-endemic and endemic regions continue to be documented in India, Nepal, and Bhutan (Hirve et al., 2017; Banjara and Joshi, 2020; Tobgay et al., 2021; Singh and Sundar, 2022). It is important to highlight that reaching the elimination target does not mean the disease is gone; it means VL is no longer a major public health problem. Even in districts that have reached the elimination threshold, the *L. donovani* parasite can still persist and the possibility of an outbreak remains if surveillance is not maintained. Further, the emerging challenge of Atypical Cutaneous Leishmaniasis (ACL) caused by *L. donovani* is on an increase in Sri Lanka, India and Nepal.

## Atypical cutaneous leishmaniasis in South East Asis: An emerging threat

The clinical manifestations of leishmaniasis were historically largely determined by the *Leishmania* species with *L. donovani/L. infantum* typically causing VL and *L. major/L. tropica* causing CL. This association between species and clinical presentation is however undergoing a paradigm shift in some regions where *L. donovani* is increasingly associated with CL, also known as Atypical Cutaneous Leishmaniasis (ACL). This trend is especially present in newer endemic sites. The characteristic features of ACL caused by *L. donovani* vs typical cutaneous leishmaniasis (CL) caused by *L. tropica* complex are largely indistinguishable. The ACL specific lesions mostly appeared as localized cutaneous skin lesions with characteristic CL lesion-specific raised borders, serous crusting and ulceration along with epidermal changes exhibiting acanthosis, papillomatosis and granulomatous inflammation with no one feature that can differentiate them. ACL/CL lesions are distinguished from PKDL that typically exhibit macular and papular rashes over different parts of the body. With this scenario, a diagnostic test that can molecularly differentiate disease specific causative parasite species/strain is required.

Molecular identification of the parasite species using species-specific PCR/PCR-RFLP initially identified genetically divergent and region-specific *L. donovani* ACL isolates from Sri Lanka, India, Nepal and Bhutan that were distinct from the VL-causing Mon-2 *L. donovani* zymodeme from Bihar India, Nepal and Bangladesh (Ranasinghe et al., 2013; Zhang et al., 2014; Kumar et al., 2015; Siriwardana et al., 2019; Bastola et al., 2020; Pal et al., 2020; Thakur et al., 2020; Tharakan et al., 2020; Rai et al., 2023). It is important to recognize that ACL represents a new *L. donovani* reservoir and that this reservoir may have the potential to evolve or revert into VL causing variants fuelling future VL outbreaks and threaten the VL elimination program advances.

Comparative genome-wide analysis of *L. donovani* isolates from VL and ACL cases from known and newer endemic sites can demonstrate the emergence of region-specific parasite genotypes, their origin and genetic relatedness. In this context, whole genome sequencing (WGS) has helped to decipher the genetic makeup of isolates from the Indian Subcontinent (ISC) as a heterogeneous *L. donovani* population with discrete genetic lineages circulating in endemic lowlands of Nepal and Bihar India (identified as the core group, CG) and a rare and emerging ISC1 lineage in the highlands of Nepal (Imamura et al., 2016; Cuypers et al., 2018; Seblova et al., 2019). More recently, isolates from VL endemic sites in western Nepal have been genetically identified as *L. donovani* sub-lineages also within the ISC1 clade (Monsieurs et al., 2024). In a neighbouring state of India, Himachal Pradesh, WGS of three *L. donovani* ACL isolates exhibit genetic heterogeneity among themselves and originate from the ISC1 clade with evidence for intraspecies hybrids and non-hybrid lineages (Lypaczewski et al., 2022, 2024). In Sri Lanka, *L. donovani* variants from ACL cases are distinct from the ISC1 clade, but are in some cases remarkably derived from interspecies hybridization between Ethiopian *L. donovani* strains with the CL causing species *L. major* and *L. tropica* (Lypaczewski and Matlashewski, 2021). Considering the emergence of these novel *L. donovani* variants such as in the ISC1 cluster in India and Nepal and the distinct Sri Lanka clusters, an evolving *L. donovani* genome is supporting the emergence of ACL endemic zones and expansion of newer mountainous niches for VL. This phenomenon underscores the urgent need to recognize the evolution of *L. donovani* as a new challenge to the VL elimination program and to public health in the affected areas.

## Discussion

The impact of the VL elimination program has been impressive with Bangladesh reaching the elimination target and the number of cases in India and Nepal dramatically reduced. Surveillance through active and passive case detection, improved case management with rapid diagnosis and effective treatments and vector control have all contributed to this success. Concomitant with this success, the occurrence of multiple clinical entities with skin manifestations associated with atypical CL caused by novel *L. donovani* variants and PKDL caused by yet different *L. donovani* strains can be misdiagnosed as typical CL caused by *L. tropica* complex. In lieu of the new outbreak areas with these disease formats, public health authorities should increase surveillance in these regions and should establish molecular diagnostics strategies to differentiate parasite species/strains associated with each type of local pathology. The evolution of novel *L. donovani* lineages in new locations including North East India, Nepalese highlands, Bhutan and Sri Lanka that cause ACL and VL is now becoming more apparent. These novel *L. donovani* lineages, such as the ISC1 lineage in newer endemic pockets in Himachal and the highlands of Nepal could eventually undermine the advances made by the VL elimination program.

A molecular surveillance strategy is necessary to follow the movement and expansion of these and other region-specific *L. donovani* lineages. Complete genome sequencing data could provide the necessary insights into parasite transmission patterns in newer geographical niches in relation to the disease phenotype. Knowledge gained will address important questions such as identifying the principal reservoir(s) for transmission and whether ACL associated *L. donovani* lineages can potentially visceralize to cause asymptomatic, VL or PKDL cases. The genome sequence surveillance data could be coupled with the re-introduction of the leishmanin skin test (LST) to determine the extend of new and previous transmission (Dey et al., 2023).This information could justify the initiation of targeted ACL elimination programs to stop the migration of this disease into more densely susceptible populations. Much has been learned from the successes of the VL elimination program and this now needs to be leveraged toward ensuring there are no major disease outbreaks of novel *L. donovani* lineages.

## Data Availability

The original contributions presented in the study are included in the article/supplementary material. Further inquiries can be directed to the corresponding author.

## References

[B1] Alvar J. Velez I. D. Bern C. Herrero M. Desjeux P. Cano J.. (2012). Leishmaniasis worldwide and global estimates of its incidence. PloS One 7, e35671. doi: 10.1371/journal.pone.0035671 22693548 PMC3365071

[B2] Banjara M. R. Joshi A. B. (2020). Evidence for visceral leishmaniasis elimination in Nepal. Lancet Glob Health 8, e161–e162. doi: 10.1016/S2214-109X(19)30538-8 31981545

[B3] Bastola A. Shrestha M. Lamsal M. Shrestha S. Prajapati S. Adhikari A.. (2020). A case of high altitude cutaneous leishmaniasis in a non-endemic region in Nepal. Parasitol. Int. 74, 101991. doi: 10.1016/j.parint.2019.101991 31520692

[B4] Cuypers B. Berg M. Imamura H. Dumetz F. De Muylder G. Domagalska M. A.. (2018). Integrated genomic and metabolomic profiling of ISC1, an emerging leishmania donovani population in the Indian subcontinent. Infect. Genet. Evol. 62, 170–178. doi: 10.1016/j.meegid.2018.04.021 29679745 PMC6261844

[B5] Dey R. Alshaweesh J. Singh K. P. Lypaczewski P. Karmakar S. Klenow L.. (2023). Production of leishmanin skin test antigen from leishmania donovani for future reintroduction in the field. Nat. Commun. 14, 7028. doi: 10.1038/s41467-023-42732-2 37919280 PMC10622560

[B6] Dorji T. Dorjee S. Wangdi T. Tshokey T. Pradhan A. R. Penjor K.. (2024). Efforts toward the elimination of visceral leishmaniasis in South Asia: A review of progress in Bhutan. Am. J. Trop. Med. Hyg 110, 633–638. doi: 10.4269/ajtmh.23-0642 38471147 PMC10993832

[B7] Epidemiology and Disease Control Division. (2019). National Guideline on Kala-azar Elimination Program (Nepal: Department of Health Services).

[B8] Hirve S. Kroeger A. Matlashewski G. Mondal D. Banjara M. R. Das P.. (2017). Towards elimination of visceral leishmaniasis in the Indian subcontinent-translating research to practice to public health. PloS Negl. Trop. Dis. 11, e0005889. doi: 10.1371/journal.pntd.0005889 29023446 PMC5638223

[B9] Imamura H. Downing T. Van Den Broeck F. Sanders M. J. Rijal S. Sundar S.. (2016). Evolutionary genomics of epidemic visceral leishmaniasis in the Indian subcontinent. Elife 5, e12613. doi: 10.7554/eLife.12613.031 27003289 PMC4811772

[B10] Kumar A. Singh V. K. Tiwari R. Madhukar P. Rajneesh Kumar S.. (2023). Post kala-azar dermal leishmaniasis in the Indian sub-continent: Challenges and strategies for elimination. Front. Immunol. 14, 1236952. doi: 10.3389/fimmu.2023.1236952 37638047 PMC10451093

[B11] Kumar N. P. Srinivasan R. Anish T. S. Nandakumar G. Jambulingam P. (2015). Cutaneous leishmaniasis caused by leishmania donovani in the tribal population of the agasthyamala biosphere reserve forest, Western Ghats, Kerala, India. J. Med. Microbiol. 64, 157–163. doi: 10.1099/jmm.0.076695-0 25480880

[B12] Leelayoova S. Siripattanapipong S. Manomat J. Piyaraj P. Tan-Ariya P. Bualert L.. (2017). Leishmaniasis in Thailand: A review of causative agents and situations. Am. J. Trop. Med. Hyg 96, 534–542. doi: 10.4269/ajtmh.16-0604 28093539 PMC5361524

[B13] Lypaczewski P. Chauhan Y. Paulini K. Thakur L. Chauhan S. Roy E. I.. (2024). Emerging leishmania donovani lineages associated with cutaneous leishmaniasis, himachal Pradesh, India 2023. Emerg. Infect. Dis. 30, 1957–1959. doi: 10.3201/eid3009.231595 39174021 PMC11346985

[B14] Lypaczewski P. Matlashewski G. (2021). Leishmania donovani hybridisation and introgression in nature: A comparative genomic investigation. Lancet Microbe 2, e250–e258. doi: 10.1016/S2666-5247(21)00028-8 35544170

[B15] Lypaczewski P. Thakur L. Jain A. Kumari S. Paulini K. Matlashewski G.. (2022). An intraspecies leishmania donovani hybrid from the Indian subcontinent is associated with an atypical phenotype of cutaneous disease. iScience 25, 103802. doi: 10.1016/j.isci.2022.103802 35198868 PMC8841885

[B16] Manomat J. Leelayoova S. Bualert L. Tan-Ariya P. Siripattanapipong S. Mungthin M.. (2017). Prevalence and risk factors associated with leishmania infection in Trang Province, Southern Thailand. PloS Negl. Trop. Dis. 11, e0006095. doi: 10.1371/journal.pntd.0006095 29155831 PMC5714378

[B17] Monsieurs P. Cloots K. Uranw S. Banjara M. R. Ghimire P. Burza S.. (2024). Source tracing of leishmania donovani in emerging foci of visceral leishmaniasis, Western Nepal. Emerg. Infect. Dis. 30, 611–613. doi: 10.3201/eid3003.231160 38407178 PMC10902524

[B18] National Center for Vector Borne Diseases Control. (2024). Kala-azar Situation in India. Available online at: https://ncvbdc.mohfw.gov.in/index1.php?lang=1&level=2&sublinkid=5945&lid=3750. (accessed September 8, 2024).

[B19] Pal A. Saha A. Chatterjee S. Saha S. (2020). A case of mutilating localized cutaneous leishmaniasis caused by leishmania donovani from Bhutan. Indian J. Dermatol. Venereol Leprol 86, 536–539. doi: 10.4103/ijdvl.IJDVL_801_19 32769303

[B20] Pandey K. Dumre S. P. Shah Y. Acharya B. K. Khanal L. Pyakurel U. R.. (2023). Forty years, (1980-2019) of visceral leishmaniasis in Nepal: Trends and elimination challenges. Trans. R Soc. Trop. Med. Hyg 117, 460–469. doi: 10.1093/trstmh/trad001 36715092

[B21] Rai T. Shrestha S. Prajapati S. Bastola A. Parajuli N. Ghimire P. G.. (2023). Leishmania donovani persistence and circulation causing cutaneous leishmaniasis in unusual-foci of Nepal. Sci. Rep. 13, 12329. doi: 10.1038/s41598-023-37458-6 37516780 PMC10387047

[B22] Ranasinghe S. Wickremasinghe R. Munasinghe A. Hulangamuwa S. Sivanantharajah S. Seneviratne K.. (2013). Cross-sectional study to assess risk factors for leishmaniasis in an endemic region in Sri Lanka. Am. J. Trop. Med. Hyg 89, 742–749. doi: 10.4269/ajtmh.12-0640 23918217 PMC3795106

[B23] Ruang-Areerate T. Ruang-Areerate P. Manomat J. Naaglor T. Piyaraj P. Mungthin M.. (2023). Genetic variation and geographic distribution of leishmania orientalis and leishmania Martiniquensis among Leishmania/HIV co-infection in Thailand. Sci. Rep. 13, 23094. doi: 10.1038/s41598-023-50604-4 38155252 PMC10754904

[B24] Seblova V. Dujardin J. C. Rijal S. Domagalska M. A. Volf P. (2019). Isc1, a new leishmania donovani population emerging in the Indian sub-continent: Vector competence of phlebotomus argentipes. Infect. Genet. Evol. 76, 104073. doi: 10.1016/j.meegid.2019.104073 31629887

[B25] Shrestha M. Khatri-Chhetri M. Poudel R. C. Maharjan J. Dumre S. P. Manandhar K. D.. (2019). Molecular evidence supports the expansion of visceral leishmaniasis towards non-program districts of Nepal. BMC Infect. Dis. 19, 444. doi: 10.1186/s12879-019-4083-3 31113385 PMC6528229

[B26] Singh O. P. Sundar S. (2022). Visceral leishmaniasis elimination in India: Progress and the road ahead. Expert Rev. Anti Infect. Ther. 20, 1381–1388. doi: 10.1080/14787210.2022.2126352 36111688

[B27] Siriwardana Y. Zhou G. Deepachandi B. Akarawita J. Wickremarathne C. Warnasuriya W.. (2019). Trends in recently emerged leishmania donovani induced cutaneous leishmaniasis, Sri Lanka, for the first 13 years. BioMed. Res. Int. 2019, 4093603. doi: 10.1155/2019/4093603 31111052 PMC6487155

[B28] Thakur L. Singh K. K. Kushwaha H. R. Sharma S. K. Shankar V. Negi A.. (2020). Leishmania donovani infection with atypical cutaneous manifestations, Himachal Pradesh, India 2014-2018. Emerg. Infect. Dis. 26, 1864–1869. doi: 10.3201/eid2608.191761 32687048 PMC7392404

[B29] Tharakan S. J. Peter Cv D. Karthik R. Rupa V. Rose W. Thomas M.. (2020). Case report: A single-center case series on skin manifestations of leishmaniasis from a non-endemic state in Southern India. Am. J. Trop. Med. Hyg 104, 928–933. doi: 10.4269/ajtmh.20-0938 33377447 PMC7941818

[B30] Tobgay T. Dorjee S. Pradhan A. Wangdi T. Drukpa T. Namgye R.. (2021). Is leishmaniasis donovani elimination feasible in Bhutan? A review of current prevention and control mechanisms in Bhutan. Bhutan Health J. 6, 27–30. doi: 10.47811/bhj.106 PMC11286285 39076348

[B31] World Health Organization. (2015). Regional Strategic Framework for Elimination of Kala-azar from the South-East Asia Region, (2005-2015) (Regional Office for South-East Asia, New Delhi, India: World Health Organization).

[B32] World Health Organization. (2021a). Ending the Neglect to Attain the Sustainable Development Goals: A Road Map for Neglected Tropical Diseases 2021–2030. Geneva.

[B33] World Health Organization. (2021b). Weekly epidemiological record 2021, vol. 96, 35 [full issue. Weekly Epidemiological Rec. = Relevé épid miologique hebdomadaire 96, 401–420.

[B34] World Health Organization. (2022). Regional Strategic Framework for Accelerating and Sustaining Elimination of kala-azar in the South-east Asia Region 2022-2026 (Regional Office for South-East Asia, New Delhi, India: World Helath Organization).

[B35] World Health Organization. (2023). Leishmaniasis. Available online at: https://www.who.int/news-room/fact-sheets/detail/leishmaniasis. (accessed September 8, 2024).

[B36] World Health Organization. (2024). Global report on neglected tropical diseases 2024 stronger together, towards 2030. (Geneva: World Health Organization).

[B37] Yajima A. Lin Z. Mohamed A. J. Dash A. P. Rijal S. (2023). Finishing the task of eliminating neglected tropical diseases (NTDs) in who South-East Asia Region: Promises kept, challenges, and the way forward. Lancet Reg. Health Southeast Asia 18, 100302. doi: 10.1016/j.lansea.2023.100302 38028173 PMC10667292

[B38] Yangzom T. Cruz I. Bern C. Argaw D. Den Boer M. Velez I. D.. (2012). Endemic transmission of visceral leishmaniasis in Bhutan. Am. J. Trop. Med. Hyg 87, 1028–1037. doi: 10.4269/ajtmh.2012.12-0211 23091191 PMC3516070

[B39] Zhang W. W. Ramasamy G. Mccall L. I. Haydock A. Ranasinghe S. Abeygunasekara P.. (2014). Genetic analysis of leishmania donovani tropism using a naturally attenuated cutaneous strain. PloS Pathog. 10, e1004244. doi: 10.1371/journal.ppat.1004244 24992200 PMC4081786

